# First Lines of Physiology

**Published:** 1838-07

**Authors:** 


Art. IV.—*First Lines of Physiology ; designed for the use of Students of Medicine.* By Daniel Oliver, M.D., Professor of the Theory and Practice of Physic in Dartmouth College.—*Boston,* 1835. 8vo. pp. 520.

We are happy to correct an error into which we fell when reviewing Dr. Dunglison's Physiology, (in stating that it was the only systematic treatise on the subject that had appeared in recent times on the other side of the Atlantic,) by noticing this volume, which has since been transmitted to us. Without the comprehensiveness of detail which Dr. D.'s work embraces, this treatise is evidently the product of no inferior research; and, although we do not agree with *all* the opinions expressed by the author in his brief but usually clear summaries of controverted questions, they are not advanced with that dogmatism which leaves the student in the belief that he is in complete possession of the subject, but rather encourage him to further enquiry by stimulating his curiosity and exciting an interest in it. The somewhat abstract style in which many parts of the volume are written, (in which respect it resembles Dr. Alison's Outlines more than any other English work on the science,) is better suited for the use of a student attending lectures in which general doctrines are explained and illustrated, than for the ordinary reader. It appears to have been Dr. Oliver's intention to effect this purpose; and we are disposed to think very favorably of the general accomplishment of it. The concluding chapter, on Animal Magnetism, is principally derived from the writings of Rostan and Georget; and we think that our author has done quite right in directing the attention of his young readers to a class of phenomena, which however explained, must be admitted to be so remarkable, and on which Laplace has thus expressed himself with the philosophic spirit which is the best consequence of high scientific attainments.

"The singular effects which result from the extreme sensibility of the nerves in certain individuals has given birth to different opinions on the existence of a new agent, which has received the name of animal magnetism. It is natural to think that the action of these causes is very feeble, and may easily be disturbed by a great variety of accidental circumstances; so that, from the fact that, in many cases, this agent has failed to manifest itself we ought not to conclude that it never exists. We are so far from being acquainted with all the agents in nature, and their different modes of action, that it would be unphilosophical to deny the existence of phenomena, merely because, in the present state of our knowledge, they are inexplicable." (p. 513.)

